# Saccades are phase-locked to alpha oscillations in the occipital and medial temporal lobe during successful memory encoding

**DOI:** 10.1371/journal.pbio.2003404

**Published:** 2017-12-21

**Authors:** Tobias Staudigl, Elisabeth Hartl, Soheyl Noachtar, Christian F. Doeller, Ole Jensen

**Affiliations:** 1 Donders Institute for Brain, Cognition and Behaviour, Radboud University, Nijmegen, The Netherlands; 2 Epilepsy Center, Department of Neurology, University of Munich, Munich, Germany; 3 Kavli Institute for Systems Neuroscience, Centre for Neural Computation, The Egil and Pauline Braathen and Fred Kavli Centre for Cortical Microcircuits, NTNU & St. Olavs Hospital, Trondheim, Norway; 4 Centre for Human Brain Health, School of Psychology, University of Birmingham, Birmingham, United Kingdom; Vanderbilt University, United States of America

## Abstract

Efficient sampling of visual information requires a coordination of eye movements and ongoing brain oscillations. Using intracranial and magnetoencephalography (MEG) recordings, we show that saccades are locked to the phase of visual alpha oscillations and that this coordination is related to successful mnemonic encoding of visual scenes. Furthermore, parahippocampal and retrosplenial cortex involvement in this coordination reflects effective vision-to-memory mapping, highlighting the importance of neural oscillations for the interaction between visual and memory domains.

## Author summary

In everyday life, we constantly move our eyes to sample visual information. In order to make the sampling efficient, these eye movements need to be coordinated with the intrinsic brain dynamics that constrain visual computations. The present study provides novel evidence for how this coordination is achieved at the neuronal level, from 2 independent data sets: direct brain recordings in epileptic patients and noninvasive magnetoencephalography recordings in healthy participants. Both studies showed that eye movements are locked to the phase of alpha oscillations—synchronous and coherent neuronal electrical activity at 7–14 Hz—just prior to a saccade, i.e., a rapid eye movement that abruptly changes the point of fixation. Importantly, this coordination is predictive of successful memory encoding.

## Introduction

Sampling of visual information has been shown to be rhythmic rather than continuous [1–3]. In particular, brain rhythms clocked by oscillations in the alpha (7–14 Hz) range [4] constrain visual sampling: electroencephalography (EEG)/magnetoencephalography (MEG) studies in humans have shown that the trial-by-trial fluctuations in near-threshold visual perception performance depend on the phase of alpha oscillations prior to stimulus presentation [5,6]. Saccadic eye movements overtly sample visual scenes. Here we ask how brain oscillations and saccades are coordinated in order to allow visual information to be encoded in memory areas.

**Fig 1:**
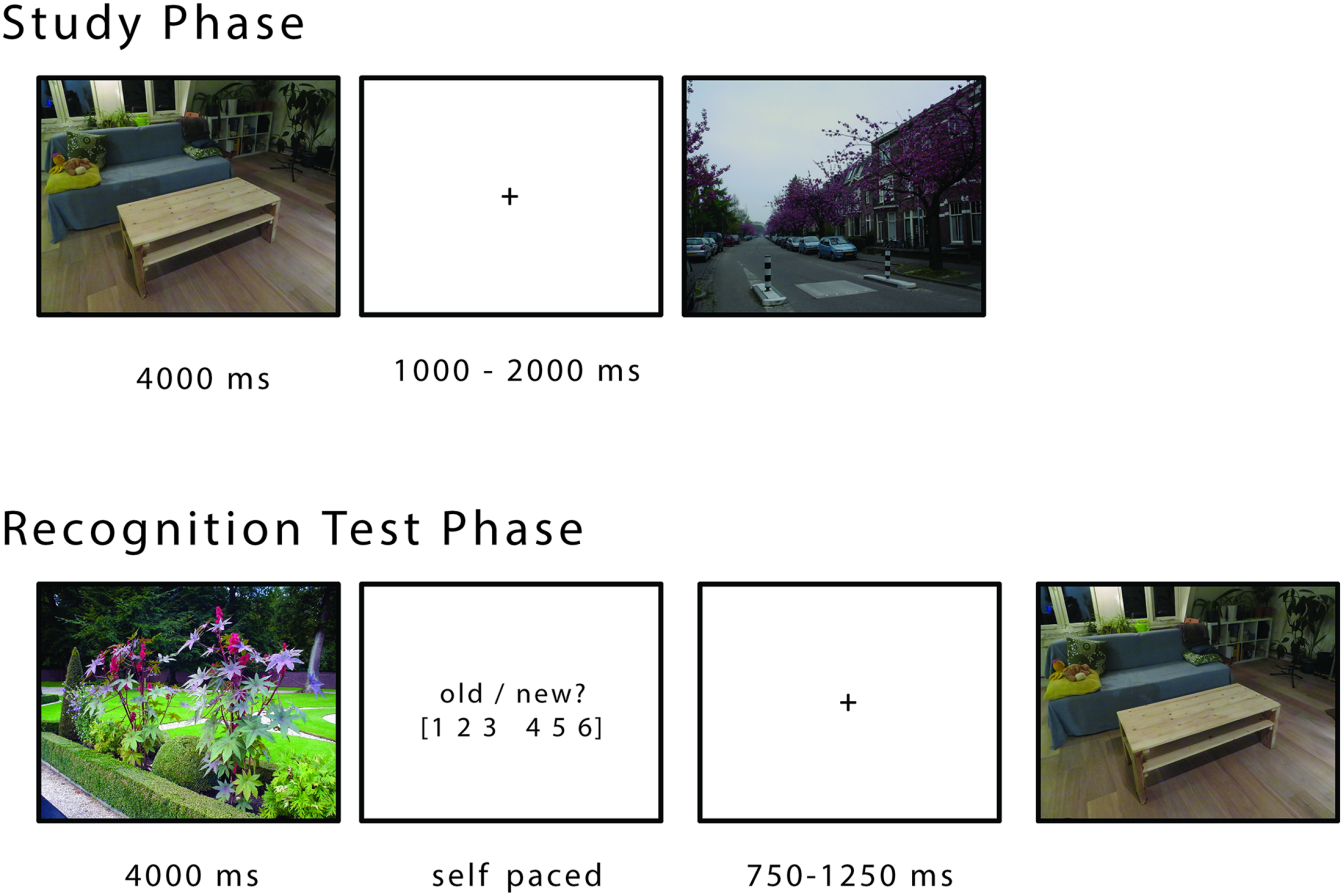
Procedure. During the study phase, participants viewed natural scenes (free viewing), indicating whether the depicted scene was indoors or outdoors (shallow encoding task). After a short distracter task (approximately 6 min), all the images from the study phase were presented again, randomly interleaved with new pictures. Participants were prompted to judge whether a given image was new or old on a 6-point scale. Image credit: Atsuko Takashima.

We addressed this question by tracking eye movements in separate memory experiments involving MEG in healthy adults and intracranial recordings in epileptic patients (Fig 1). Participants were asked to remember images of visual scenes, and we later probed their memory. The phase locking [7] between presaccadic brain oscillations in relation to saccade onset was contrasted between later-remembered and later-forgotten images. Building on prior evidence on the cortical origins of alpha activity underlying visual information sampling [8,9], we hypothesized that higher phase locking in occipital lobe would be related to successful memory performance. MEG and intracranial data both showed that eye movements are locked to the phase of alpha oscillations prior to a saccade. Importantly, this coordination was related to successful memory encoding.

## Results

### Saccade-related phase locking

**Fig 2:**
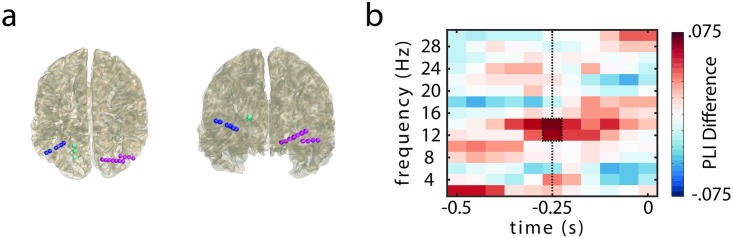
Presaccadic phase locking in occipital leads of depth electrodes. (A) Electrode locations of depth electrodes in 3 epilepsy patients (color coded). (B) Phase-locking difference (later remembered–later forgotten) in the occipital leads of the depth electrodes prior to saccade onset (t = 0 s). Significantly higher phase locking in later-remembered versus later-forgotten trials; cluster highlighted by black box (*p* < 0.005, 2-sided test, fixed-effects statistics, 15 leads in bipolar montage). The data set used to generate the analyses shown in this figure can be found here: https://osf.io/tpykv. PLI, phase-locking index.

**Fig 3:**
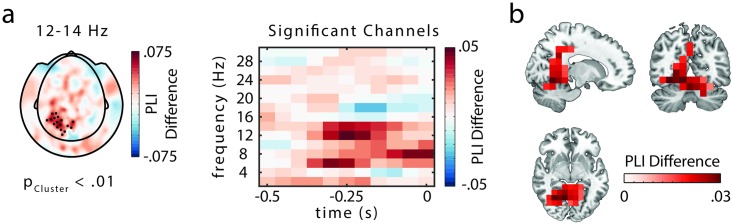
Presaccadic phase locking in the magnetoencephalography (MEG) data. (A) MEG sensor (planar gradients) analysis shows significantly higher phase locking (phase-locking index [PLI]) for later-remembered trials than for later-forgotten trials at 12–14 Hz, based on intracranial results (*p* < 0.01, 2-sided test, significant sensors highlighted). The time-frequency representation of the phase-locking difference averaged across highlighted sensors shows the peak of the difference to be at 250 ms prior to saccade onset (t = 0 s). (B) Phase-locking analysis (PLI) at source level using a dynamic imaging of coherent sources (DICS) beamformer approach. Cluster-based permutation statistics indicated a significant difference between later-remembered and later-forgotten trials (*p* < 0.05, 2-sided test). While the maximum difference was in parahippocampal areas, the source extended to visual, parietal, and temporal areas. The data set used to generate the analyses shown in this figure can be found here: https://osf.io/tpykv.

In order to investigate the temporal coordination of saccades and brain oscillations, the time-frequency representations of phase and power of the MEG and intracranial data were aligned to saccade onsets. Accordingly, high presaccadic phase locking would demonstrate an effective coordination of saccades in relation to brain oscillations. The intracranial data recorded from 3 patients with occipital depth electrodes (Fig 2A) revealed a significantly higher phase locking for later-remembered as compared to later-forgotten trials in the alpha band (12–14 Hz, cluster randomization: *p* < 0.005, controlling for multiple comparisons over frequencies, 2-sided test, fixed-effects statistics). Fig 2B depicts a time-frequency representation of the difference in phase locking, indicating that the effect is centered around 250 ms prior to saccade onset at 12–14 Hz. When aligning the data to saccade offset (i.e., fixation onset), no significant differences in phase locking were found (presaccade: *p* > 0.21, S1A Fig; postsaccade: *p* > 0.25, S1B Fig), in line with the idea that activity timed to saccade onset is important for visual processing [10]. The intracranial results then guided the analyses in the group-level study by confining the frequency of interest to 12–14 Hz, where MEG data here is presented from 22 healthy participants performing the memory task (see “Materials and methods” for exclusion criterions). A cluster-based permutation test revealed a significant difference in presaccade phase locking between later-remembered and later-forgotten images in the alpha band (12–14 Hz; cluster randomization: *p* < 0.01, controlling for multiple comparisons over sensors, 2-sided test, Fig 3A).

In the posterior sensors forming a cluster, the difference was most pronounced approximately 250 ms prior to saccade onset (Fig 3A). Unfiltered data from exemplar depth electrodes and MEG sensors depicting saccade-onset locked potentials are shown in S2 Fig. Analyzing the phase locking for later-remembered and later-forgotten pictures separately suggested the existence of a preferred alpha phase for later-remembered, but not later-forgotten, trials (S3 Fig).

Additional analyses of presaccadic spectral power indicated that the phase-locking results were not biased by spectral power (S4 Fig). A control analysis, in which we related phase locking to stimulus onset (irrespective of saccadic eye movements), revealed no significant differences between later-remembered and later-forgotten scenes (S8 Fig). However, when analyzing power after stimulus onset (irrespective of saccadic eye movements), significantly less alpha power was found for remembered as compared to forgotten scenes (12–14 Hz; cluster-randomization: *p* < 0.019, controlling for multiple comparisons over sensors and time, 2-sided test, S8 Fig), highlighting the difference between stimulus-onset-related subsequent memory studies (for an overview, see [11]) and the present saccade-related phase-locking analyses during free viewing. No difference was found when analyzing phase locking or power after saccade onset (S9 Fig).

Since the average fixation duration between typical eye movements is less than 500 ms, we also analyzed saccades with a minimum fixation duration of 200 ms prior to saccade onset. In this analysis, significantly higher phase locking for later-remembered trials than for later-forgotten trials at 10 Hz was found (cluster randomization: *p* < 0.05, controlling for multiple comparisons over sensors, 2-sided test, S5 Fig). Note that the shorter time window of 200 ms is at the expense of frequency resolution now being approximately 5 Hz). Because this analysis produced higher trial numbers, data from all 36 participants could be analyzed. Again, significantly higher phase locking for later-remembered trials than for later-forgotten trials was found (cluster randomization: *p* < 0.005, controlling for multiple comparisons over sensors, 2-sided test, S5 Fig). We conclude that the memory encoding related to saccades phase locked to alpha oscillations is robust with respect to presaccadic epochs of different lengths.

In order to identify the sources of the effects, we computed phase locking in the alpha band for virtual sensors, applying a dynamic imaging of coherent sources (DICS) beamformer [12]. Cluster-based permutation statistics at the source level yielded a significantly higher phase-locking index for later-remembered trials than for later-forgotten trials (*p* < 0.01, 2-sided test; Fig 3B). The cluster spanned from visual to parietal and temporal areas, extending into the cerebellum (Fig 3B). The largest differences were found in the parahippocampal gyrus and the retrosplenial cortex, which have been shown to support the encoding of visual scenes [13–15], and extended into the posterior hippocampus. The MEG source localization is supported by intracranial data from parahippocampal depth electrodes in the 3 patients, showing significantly higher phase locking for later-remembered trials versus later-forgotten trials in the alpha range (8–10 Hz, *p* cluster < 0.05; 2-sided test, fixed-effects statistics; S6 Fig)

### Memory performance and saccade metrics

The memory performance of the 22 participants included in the main MEG analyses (d-prime = 2.13, SE = 0.11) was considerably higher than the memory performance in patients (d-prime = 0.79). In total, 38,177 saccades were detected in the eye tracking data (22 participants, mean = 1,735.3, SE = 83.6), resulting in an average saccade rate of 2.30 Hz (SE = 0.11). The saccade rate is at the lower end of the typically reported range, which can be partly explained by the use of conservative saccade detection criterions to exclude ambiguous eye tracking data. The saccade rate was significantly higher (t_21_ = 7.34, *p* = 3.17 * 10^−7^) for later-remembered (mean = 2.37 Hz, SE = 0.10) versus later-forgotten (mean = 2.01 Hz, SE = 0.11) scenes, which has been reported previously [16,17], but see [18] for conflicting evidence. The average saccade duration was 28.8 ms (SE = 0.6), and the average fixation duration was 342.5 ms (SE = 13.8). Saccade directions displayed a horizontal bias but were not different for later-remembered versus later-forgotten trials (Kuiper 2-sample test for each participant, all *p*-values > 0.1; S7 Fig).

When only events with a minimum fixation period of 500 ms prior to saccade onset were included (as in the main analyses), 3,837 saccades remained (mean = 174.4, SE = 13.26) in the eye tracking data, resulting in an average saccade rate of 0.36 Hz (SE = 0.01). There was no significant difference between saccade rates for later-remembered (0.22, SE = 0.02) and later-forgotten (0.25, SE = 0.02) scenes (t_21_ = −1.77, *p* = 0.091), indicating that the subsequent memory effect found in all saccades (above) cannot be generalized across all types of saccades. The average saccade duration was 28.2 ms (SE = 0.8), and the mean fixation duration was 802.9 ms (SE = 20.4). Saccade directions displayed a horizontal bias but were not different for later-remembered versus later-forgotten trials (Kuiper 2-sample test for each participant, all *p*-values > 0.1; S7 Fig).

In the intracranial data, a total of 1,415 saccades were detected (3 participants, mean = 471.7), resulting in an average saccade rate of 1.25 Hz and a mean fixation duration of 474 ms. Note that this low saccade rate can partly be explained by the fact that electrooculography (EOG) signals were used to detect saccades in patients, which is less sensitive than eye tracking, and by conservative saccade detection criterions to exclude ambiguous EOG data. The mean saccade duration in patients was 33.7 ms. The saccade rate for later-remembered scenes (mean = 1.1551) was lower than for later-forgotten scenes (mean = 1.2906) for the patient data.

When only events that were free of saccades and blinks in a 0.5-s interval prior to saccade onset were included (as in the main analyses), 434 saccades remained (mean = 144.7) in the EOG data, resulting in an average saccade rate of 0.42 Hz. The saccade rate for later-remembered scenes (mean = 0.44) was similar to that of later-forgotten scenes (mean = 0.40) for the patient data. The average saccade duration was 26.2 ms, and the mean fixation duration was 878.6 ms.

## Discussion

In 2 independent data sets, we provide novel evidence for a functionally relevant coordination of saccadic eye movements and brain activity. Both the intracranial and the MEG data show that retinal inputs are temporally aligned to a preferential alpha phase. Importantly, this coordination was related to successful memory encoding, suggesting a mechanistic role for alpha oscillations in coordinating the encoding of visual information. Furthermore, our results point to an active involvement of task-relevant brain areas in this coordination: MEG and intracranial data yielded the occipital cortex, the parahippocampal gyrus, and the retrosplenial cortex as sources of the coordination of saccades and alpha phase, which have been shown to support the encoding of visual scenes [13–15]. The engagement of scene-selective areas may reflect effective vision-to-memory mapping along visual, parietal, and posterior temporal cortices [19].

Our findings are in line with work from the 1960s [20] suggesting a relationship between alpha oscillations and saccades; however, this effect was not related to perception and memory. They also support the notion of a preferred alpha phase for the execution of eye movements [21], by suggesting that during optimal information encoding, the execution of saccades is on hold until the end of an alpha duty cycle. We propose that effective coordination of saccades and brain oscillations allows for optimizing the speed of processing in the visual system [22]. The intracranial data in occipital and parahippocampal electrodes showed enhanced phase locking in the alpha band for later-remembered trials as compared to later-forgotten trials, albeit with the frequencies being slightly lower in the parahippocampal (8−10 Hz) than in the occipital depth electrodes (12−14 Hz). This could be interpreted as a shift in the dominant frequency of brain areas along the hierarchy, from visual to memory areas.

The main results presented here rely on events with a minimum fixation duration of 500 ms prior to saccade onset. The upside of this selection is the exclusion of other saccades or blinks that would contaminate the time window of interest while keeping a reasonable frequency resolution. On the downside, these events may not reflect stereotypical eye movement behavior, which display an average fixation duration of approximately 250 to 300 ms. However, analyzing events with a minimum fixation duration of 200 ms (at the expense of frequency resolution) showed very similar phase-locking effects, thus underscoring the robustness of our core findings.

Although memory studies often treat eye movements as artifacts, their interaction with memory processes has gained recent interest in the field [23,24]. Importantly, investigating naturalistic behavior in free-viewing paradigms, as used in the present study, has been shown to provide crucial insight into the interaction of eye movement behavior and memory processes, as, for example, relationships between visual sampling and recognition memory performance [16,17] or hippocampal blood oxygen level-dependent (BOLD) activity [25]. Going beyond these prior findings, the present results indicate that eye movements already have an effect on memory performance at the stage of their initiation, depending on their coordination with brain rhythms implicated in the sampling of visual information.

The increase in memory encoding with saccades locked to alpha phase might be supported by anticipatory attentional deployment [26]. The fact that the phase-locking difference was found prior to saccade onset might suggest planning of the upcoming to-be-attended location [27], resulting in a stronger locking between saccades and the phase of the alpha oscillation and ultimately improved memory encoding. The present results highlight the necessity for a coordination of alpha oscillations and eye movements for optimal memory encoding. Efficiently sampled visual information could then be integrated by the hippocampal memory system. A recent nonhuman primate study demonstrated that saccades were aligned to hippocampal oscillations of approximately 10 Hz [28]. Future studies should explore interregional synchronization in relation to oculomotor behavior, visual information sampling, and memory.

## Materials and methods

### Ethics statement

All participants gave written informed consent before the start of experiment in accordance with the Declaration of Helsinki. The study was approved by the local ethics committee (commission for human related research CMO-2014/288 region Arnhem/Nijmegen NL). The patients, who volunteered to participate in the study, had depth electrodes implanted for diagnostic reasons. The patients gave written informed consent. The study was approved by the ethics committee of the University of Munich.

### Participants

For the MEG part, 36 young healthy adults were included in the study. Initially, 48 participants were recruited; however, 12 were removed because of not completing the study (7 participants), excessive movement artifacts (2 participants), or technical problems during the recordings (3 participants). The 36 participants included in this study (24 females; mean age 23.1 y, range 18−30 y; 35 right handed) reported no history of neurological and/or psychiatric disorders and had normal or corrected-to-normal vision.

Additionally, 3 male patients (age range 30−60 y) with a history of drug-resistant epilepsy were recruited from the Epilepsy Center, Department of Neurology, University of Munich, Germany.

### Design, procedure, and materials

The study design comprised an MEG and an fMRI (not reported here) session. Session order was counterbalanced across participants. For each session, 3 stimulus sets of 100 photographs each were constructed. Half of the pictures depicted indoor scenes, the other half outdoor scenes (exemplary scenes are shown in Fig 1). Pictures were presented in the MEG chamber on a 39 × 46 cm back-projection screen subtending a visual angle of approximately 27° × 32°. Out of the 3 sets, 2 sets (200 scenes) were presented during encoding. During test, these 2 sets were presented again, plus the third set (100 scenes as foils). Assignment of a set to encoding or test was counterbalanced across participants. Nine additional scenes were presented during a short practice session before encoding and test in order to explain the task. Participants were made aware about the memory test before the start of the experiment.

Fig 1 illustrates the experimental procedure. At study, the pictures were presented for 4 s in random order with the constraint that no more than 4 scenes of the same type (indoor/outdoor) were shown consecutively. The participants were instructed to judge whether the depicted scene was indoors or outdoors by button press during the fixation cross. This encoding task was chosen to ensure attention to each scene and promote encoding of the images. Participants freely viewed the scenes; i.e., they were not expected to fixate. A fixation cross with variable duration (1–2 s) followed each scene.

The study phase was followed by a distracter phase during which the participants solved simple mathematical problems for approximately 1 min, underwent approximately 5 min of fixation to different locations on the screen used to evaluate eye tracker accuracy, and spent approximately 1 min with eyes open and approximately 1 min with eyes closed. The distracter phase prevented participants from covert rehearsing. The distracter period was followed by the memory test. At test, the 200 pictures from the study phase and 100 new pictures (foils) were presented for 4 s each. The presentation order was randomized, with the constraint that no more than 4 scenes of the same type (old/new) were shown consecutively. After each scene, participants were prompted to indicate their confidence on whether the scene was old or new using a 6-point response scale, ranging from “very sure old” (1) to “very sure new” (6). This picture of the rating scale remained until the participants responded. Before the next scene, a fixation cross with variable duration (750–1,250 ms) was presented. The procedure for the patients with intracranial electrodes deviated slightly (see below).

### MEG acquisition and preprocessing

MEG was recorded using a 275 whole-brain axial gradiometer system (VSM MedTech/CTF MEG, Coquitlam, Canada) installed in a magnetically shielded room. The data were sampled at 1,200 Hz following a low-pass antialiasing filter with a cutoff at 300 Hz. Additionally, horizontal and vertical electro-oculograms were recorded from bipolar Ag/AgCl electrodes (<10kΩ impedance) placed below and above the left eye and at the bilateral outer canthi. To track the position of the head during MEG recording, we used 3 head coils placed at anatomical landmarks (nasion and both ear canals). Using a real-time head localizer [29], the position of the head relative to the MEG helmet was tracked. Each participant’s nasion, left and right ear canal, and head shape were digitized with a Polhemus 3Space Fasttrack.

Preprocessing of the data was done using the Fieldtrip toolbox [30]. Data were divided into single epochs ranging from 0 to 4 s after picture onset. Epochs were corrected for cardiac artifacts using independent component analysis (ICA) and sorted according to the behavioral performance of each participant’s confidence judgments during the recognition test phase. Pictures that were confidently judged as old (responses 1, 2, and 3) constituted later-remembered scenes, and the remaining pictures were classified as later-forgotten scenes.

### Eye tracking acquisition, analyses, and trial definition

An Eyelink 1000 (SR Research) eyetracker was used to monitor the horizontal and vertical movements of the participant’s left eye. Before recording, the eye tracker was calibrated by collecting gaze fixation samples from known target points to map raw eye data on screen coordinates. Participants fixated on 9 dots sequentially on a 3-by-3 grid. After the calibration run, a validation run was performed during which the difference between current gaze fixations and fixations during the calibration was obtained. The calibration was only accepted if this difference was smaller than 1° of visual angle.

Eye tracking and MEG data were simultaneously recorded and analyzed using the Fieldtrip toolbox. Vertical and horizontal eye movements were transformed into velocities. Velocities exceeding a certain threshold (velocity > 6× the standard deviation of the velocity distribution, duration > 12 ms, see Engbert and Kliegl [31]) were defined as saccades. Saccade onsets during stimulus presentations in the study phase defined the events of interest (trials). To avoid potential artifacts from other eye movements and provide a reasonable frequency resolution of 2 Hz, only events that were free of saccades and blinks in a 0.5-s interval prior to saccade onset (i.e., a minimum fixation period of 500 ms) were included. Saccades that occurred during the presentation of scenes that were subsequently judged as old (responses 1, 2, and 3) constituted later-remembered trials. Saccades that occurred during scenes that were subsequently judged as new (responses 4, 5, and 6) constituted later-remembered trials.

After excluding all participants that had less than 30 remaining trials per condition (later remembered or later forgotten), 22 participants were included in the further analyses (3,837 trials in total, mean = 174.4, SE = 13.26; mean number of remembered trials = 109.7, SE = 8.8; mean number of forgotten trials = 64.7, SE = 9.7). In order to display the temporal dynamics of the phase locking, the trials were zero-padded to a length of 1.5 s (i.e., adding 500 ms of zeros before and after the 500 ms of data). Since typical eye movements occur approximately every 250–300 ms, the events with a minimum fixation period of 500 ms may not be representative. Therefore, we conducted additional phase-locking analysis on events with a minimum fixation period of 200 ms prior to saccade onset, including approximately 66% of all detected saccades. In the 22 participants, a total of 25,077 saccades (mean = 1,139.9, SE = 49.6; mean number of remembered trials = 802.7, SE = 57.5, mean number of forgotten trials = 337.1, SE = 26.5) were included. Since this analysis produced higher trial numbers, data from all 36 participants could be analyzed (total number of saccades = 43,226; mean = 1,201.8, SE = 41.3; mean number of remembered trials = 918.7, SE = 49.6, mean number of forgotten trials = 282.9, SE = 23.1). These trials were zero-padded to a length of 0.6 s (i.e., adding 200 ms of zeros before and after the 200 ms of data).

### Phase and power analysis

The frequency spectra of the phase and the power of the data were computed by applying a Fourier transformation to the 500 ms of data prior to saccade onset in each event, after multiplication with a hanning taper. Phase and power were calculated for frequencies between 2 and 30 Hz in steps of 2 Hz. The frequency spectra of the phase and the power were used to statistically test differences between conditions (see “Statistics”).

Synthetic planar gradient representations were approximated by relating the field at each sensor with its neighbors’ [32]. On each of the resulting 2 orthogonal gradients, Fourier coefficients were normalized by their amplitude, and the phase-locking index (PLI) [7] was calculated, by extracting the length of the resulting vector after averaging the phase angles:

$${PLI}_{tf}\;=\;|n^{-1}\sum_{r\;=\;1}^{n}e^{{ik}_{tfr}}\;|,$$

where *n* = number of trials, and *e*^*ik*^ equals the complex polar representation of phase angle *k* in trial *r*, for time-frequency point *tf*.

This was done for later-forgotten and later-remembered trials. The PLI quantifies the consistency of phases across trials at each given time-frequency point. To control for a bias in PLI due to different trial numbers in conditions, a sample of trials from the condition with the larger number of trials was randomly drawn, with the number of trials in this sample being equal to the number of trials in the condition with less trials. The PLI for this sample was computed. After repeating this procedure 1,000 times, PLI values were averaged. This average reflects an unbiased estimate of the PLI for all trials in the respective condition. After this step, the 2 planar gradients were combined.

In order to depict the temporal dynamics of phase and power in the data, time-frequency representations were computed by a sliding time window approach with a window length of 0.5 s in steps of 50 ms across the zero-padded data. After multiplying a hanning taper to each window, the Fourier transformation was calculated for frequencies between 2 and 30 Hz in steps of 2 Hz.

To identify potential confounds due to differences in spectral power, power was calculated on synthetic planar gradients, using the same approach as outlined above. Instead of computing the PLI, power values were calculated from the Fourier coefficients (amplitude squared).

The PLI analysis on events with a minimum fixation period of 200 ms prior to saccade onset was performed as defined above, with the exception that the window length was 200 ms in the sliding time window approach. Due to the resulting frequency resolution of 5 Hz, phase information was extracted from 5 to 30 Hz in steps of 5 Hz.

### Source-level analyses

To identify PLI differences in source space, a virtual sensor approach applying frequency-domain adaptive spatial filtering (DICS beamformer [12]) was implemented. This algorithm constructs a spatial filter for each specified location (each grid point; 10-mm^3^ grid). The cross spectral density for the construction of the spatial filter was calculated for the frequency of interest (12–14 Hz, using orthogonal Slepian tapers around a center frequency of 13 Hz with spectral smoothing of +/− 2 Hz), for all trials (common filter approach).

Individual structural MR images, acquired on a 3T Siemens Magnetom Prisma MRI system (Siemens, Erlangen, Germany), were aligned to the MEG coordinate system, utilizing the fiducials (nasion, left and right preauricular points) and individual head shapes recorded after the experiment. A realistic single-shell brain model [33] was constructed for each participant, based on the structural MRIs. The forward model for each participant was created using a common dipole grid (10-mm^3^ grid) of the grey matter (derived from the anatomical automatic labeling atlas [34]) volume in MNI space warped onto each participant’s anatomy. The Fourier data were projected into source space by multiplying them with the spatial accordant filters, allowing for the phase to be estimated. The PLI was computed on the 2 orientations of the source model, and later averaged, for later-remembered and later-forgotten trials, respectively.

### Statistics

Statistics followed a 2-step approach: first, differences in the intracranial data’s phase locking (later-remembered versus later-forgotten trials) were evaluated in a fixed-effect manner, by concatenating all electrodes from all patients. Cluster-based nonparametric permutation statistics [35] identified continuous frequency clusters with significant differences between later-remembered and later-forgotten PLI while controlling for multiple comparisons over frequencies. Only the cluster with the largest summed value was considered and tested against the permutation distribution. The null hypothesis that later-forgotten and later-remembered trials showed no difference in PLI was rejected at an alpha level of 0.05 (2-tailed).

Second, statistical quantification of the MEG sensor-level data was performed by a cluster-based nonparametric permutation approach [35], identifying clusters of activity on the basis of rejecting the null hypothesis while controlling for multiple comparisons over sensors. The frequency range (12–14 Hz) for the sensor-level statistics was restricted to the outcome of the intracranial data analyses. For each sensor, a test statistic was calculated, based on a paired samples *t* test comparing the PLI for later-remembered versus later-forgotten trials. Sensors showing a significant effect (*p* < 0.05, 2-sided *t* test) were clustered based on spatial adjacency, with a minimum of 2 adjacent sensors required for forming a cluster. T-statistics were summed in each cluster. Again, only the cluster with the largest summed value was considered and tested against the permutation distribution. The null hypothesis that later-forgotten and later-remembered trials showed no difference in PLI was rejected at an alpha level of 0.05 (2-tailed).

Statistical quantification of the source-level data was also performed by a cluster-based nonparametric permutation approach, now considering the clustering in voxel space. The frequency range for the source-level statistics was defined by the outcome of the sensor-level statistics, and the alpha level was set to 0.05 (2-tailed). Cluster-based nonparametric permutation statistics [35] identified continuous spatial clusters with significant differences between later-remembered and later-forgotten PLI while controlling for multiple comparisons over voxels. Only the cluster with the largest summed value was considered and tested against the permutation distribution. The null hypothesis that later-forgotten and later-remembered trials showed no difference in PLI was rejected at an alpha level of 0.05 (2-tailed).

Condition-specific PLIs for later-remembered and later-forgotten trials, separately (see S3 Fig), at the time and frequency of interest (12–14 Hz, −0.25 ms) were statistically quantified by comparing them to a distribution of surrogate PLI values. The surrogate PLI distribution was constructed for each participant and condition by shifting the data points in each condition’s trial circularly along the time axis with a random lag, for each sensor. PLI values were computed as explained above, for 1,000 random shifts. Subsequently, 10,000 surrogate grand averages were constructed by randomly drawing 1 PLI value from each participant’s surrogate distribution for each surrogate grand average. Condition-specific PLI grand averages were compared to these 10,000 surrogate grand averages on each sensor and considered to be significant if they were larger (or smaller) than 97.5% (or 2.5%) of the values in the surrogate grand average values (2-sided test).

### Intracranial data

Three male patients (age range 30–60 y) with occipital depth electrodes were included in the study. The patients had a history of drug-resistant focal epilepsy and were implanted for diagnostic reasons. Recordings were performed at the Epilepsy Center, Department of Neurology, University of Munich, Germany. The patients gave written informed consent. The procedure and design of the study was identical to the MEG procedure and design (see above), with the exception that only 100 pictures were presented during study and 200 scenes (100 old and 100 new) were presented during the memory test. This was done to compensate for inferior memory performance in a clinical setting.

Patient 1 had 10 depth electrodes implanted, covering bilateral temporal, parietal, and frontal regions and left occipital regions. Patient 2 had 10 depth electrodes implanted, covering right temporal, parietal, and occipital regions. Patient 3 had 11 depth electrodes implanted, covering left frontal, temporal, parietal, and occipital regions. The locations of the electrodes were determined using coregistered preoperative MRIs and postoperative CTs. Electrode locations were converted to MNI coordinates. Intracranial EEG was recorded from Spencer depth electrodes (Ad-Tech Medical Instrument, Racine, Wisconsin, United States) with 4–12 contacts each, 5 mm apart. Data were recorded using XLTEK Neuroworks software (Natus Medical, San Carlos, California, US) and an XLTEK EMU128FS amplifier, with voltages referenced to a parietal electrode site (1,000 Hz sampling rate). All electrodes that either were identified as located in the seizure onset zone or showed interictal spiking activity were excluded from analyses. Data were rereferenced offline to each contact’s neighboring contact (bipolar montage). All bipolar electrodes with both contacts in the occipital cortex were included in the analyses. Additionally, horizontal and vertical eye movements were recorded from bipolar Ag/AgCl electrodes (<10kΩ impedance) placed below and above the left eye and at the bilateral outer canthi.

Study phase data were cut into single epochs, ranging from 0 to 4 s after picture onset. Saccade onsets were extracted from EOG recordings using the method described above (see “Eye tracking acquisition, analyses, and trial definition”). Saccade onsets during stimulus presentations defined the events of interest (trials). All trials were visually inspected for artifacts (e.g., epileptiform spikes). Contaminated trials were excluded from the analyses. The encoding trials were sorted according to each participant’s confidence judgments during the test phase. Pictures that were confidently judged as old (responses 1, 2, and 3) constituted hits, and the remaining pictures were classified as misses. Time-frequency analyses, PLI, and statistics were computed as described above.

## Supporting information

S1 Fig Pre- and postsaccade phase locking locked to saccade offset. (A) Phase-locking difference (later remembered–later forgotten) on occipital depth electrodes prior to saccade offset (time = 0 s). There was no significant difference in phase locking between later-remembered and later-forgotten trials (*p* > 0.21; 2-sided test, fixed-effects statistics, 15 contacts in bipolar montage, time = −250 ms). (B) Phase-locking difference (later remembered–later forgotten) on occipital depth electrodes after saccade offset (time = 0 s). No significant difference in phase-locking between later-remembered and later-forgotten trials (*p* > 0.25; 2-sided test, fixed-effects statistics, 15 contacts in bipolar montage, time = 250 ms). Note that the statistical tests were performed on the center time bin (−250 ms and 250 ms, respectively). The data set used to generate the analyses shown in this figure can be found here: https://osf.io/tpykv.
(TIF)

S2 Fig Presaccadic unfiltered data. Unfiltered data, averaged across trials locked to saccade onset (time = 0 s). (A) Exemplar data from an occipital depth electrode (bipolar montage). (B) Exemplar data from a parahippocampal depth electrode (bipolar montage). (C) Exemplar data from a magnetoencephalography (MEG) sensor. Note the more regular, slow frequency fluctuations in the averaged potentials for later-remembered trials (left) as compared to later-forgotten trials (right). The data set used to generate the analyses shown in this figure can be found here: https://osf.io/tpykv.
(TIF)

S3 Fig Condition-specific phase-locking index (PLI) contrasts. (A) Difference between later-remembered trials and surrogate data at 12–14 Hz, −250 ms. (B) Difference between later-forgotten trials and surrogate data at 12–14 Hz, −250 ms. Significant sensors are highlighted (*p* < 0.05, 2-sided). The data set used to generate the analyses shown in this figure can be found here: https://osf.io/tpykv.
(TIF)

S4 Fig Presaccade power. (A) Topography of magnetoencephalography (MEG) sensor-level statistics (planar gradients) for power averaged over 12–14 Hz at −0.25 s (corresponding to the phase-locking index [PLI] effect); no significant difference (cluster-based permutation statistic, no clusters found). The bar plot (error bars represent SEM; dots indicate individual participants) depicts 12–14 Hz power on sensors showing the significant phase-locking effect. No significant difference (t_21_ = 0.07, *p* > 0.9, 2-sided *t* test) between later-remembered and later-forgotten trials in the respective time interval (−0.25 s) was found. (B) Occipital depth electrodes showed no significant difference in power (t_14_ = 1.27, *p* > 0.2, 2-sided *t* test), averaged over 12–14 Hz at −0.25 s (c) Parahippocampal depth electrodes showed no significant difference in power (t_10_ = 0.71, *p* > 0.7, 2-sided *t* test), averaged over 8–10 Hz at −0.25 s. The data set used to generate the analyses shown in this figure can be found here: https://osf.io/tpykv.
(TIF)

S5 Fig Presaccadic phase locking for 200-ms minimum fixation duration. Magnetoencephalography (MEG) sensor (planar gradients) analysis shows significantly higher phase locking (phase-locking index [PLI]) for later-remembered than forgotten trials at 10 Hz (corresponding to a frequency range of 7.5 to 12.5 Hz), averaged in the −0.2- to 0-s interval prior to saccade onset (left: *N* = 22, *p* < 0.05, 2-sided test, significant sensors highlighted; right: *N* = 36, *p* < 0.005, 2-sided test, significant sensors highlighted). The data set used to generate the analyses shown in this figure can be found here: https://osf.io/tpykv.
(TIF)

S6 Fig Presaccade phase locking on parahippocampal leads of depth electrodes. (A) Electrode locations of parahippocampal depth electrodes in 3 participants (color coded). (B) Phase-locking difference (later remembered–later forgotten) on parahippocampal depth electrodes prior to saccade onset (time = 0). Significantly higher phase locking in later-remembered versus later-forgotten trials at 8–10 Hz (*p* < 0.05, highlighted; 2-sided test, fixed-effects statistics, 11 contacts in bipolar montage). The data set used to generate the analyses shown in this figure can be found here: https://osf.io/tpykv.
(TIF)

S7 Fig Saccade directions. Saccade directions for (A) all detected saccades in the magnetoencephalography (MEG) data set and (B) for saccades in the MEG data set with a minimum fixation period of 500 ms prior to saccade onset (as used in the main analysis). For both (A) and (B), the distributions for later-remembered (middle column) and later-forgotten trials (right column) did not differ significantly (Kuiper 2-sample test for each participant, all *p*-values > 0.1). The data set used to generate the analyses shown in this figure can be found here: https://osf.io/tpykv.
(TIF)

S8 Fig Phase locking and power after stimulus onset. (A) Cluster-based permutation statistics showed no significant differences in phase locking between later-remembered and later-forgotten scenes at 12–14 Hz, controlling for multiple comparisons over time (0 to 4 s) and sensors (*p* cluster > 0.9). There were also no significant differences after averaging time over 1-s bins (all clusters *p* > 0.64). (B) Cluster-based permutation statistics (controlling for multiple comparisons over sensors and time: 0 to 4 s) showed significantly lower power for later-remembered scenes than for later-forgotten scenes at 12–14 Hz (*p* cluster < 0.018; time = 1.55–2.6 s). The data set used to generate the analyses shown in this figure can be found here: https://osf.io/tpykv.
(TIF)

S9 Fig Phase locking and power after saccade onset. (A) Topography of magnetoencephalography (MEG) sensor-level statistics (planar gradients) for phase locking averaged over 12–14 Hz at 250 ms; no significant difference (cluster-based permutation statistic, all clusters *p* > 0.72). (B) Topography of MEG sensor-level statistics (planar gradients) for power averaged over 12–14 Hz at 250 ms; no significant difference (cluster-based permutation statistic, no clusters found). The data set used to generate the analyses shown in this figure can be found here: https://osf.io/tpykv.
(TIF)

## Abbreviations

BOLD: blood oxygen level-dependent
DICS: dynamic imaging of coherent sources
EEG: electroencephalography
EOG: electrooculography
MEG: magnetoencephalography
PLI: phase-locking index
