# Supplementary figures and images for: Saccades are phase-locked to alpha oscillations in the occipital and medial temporal lobe during successful memory encoding
Source: PLoS Biol. 2017 Dec 21;15(12):e2003404. doi: 10.1371/journal.pbio.2003404 (PMC5766246; doi:10.1371/journal.pbio.2003404)

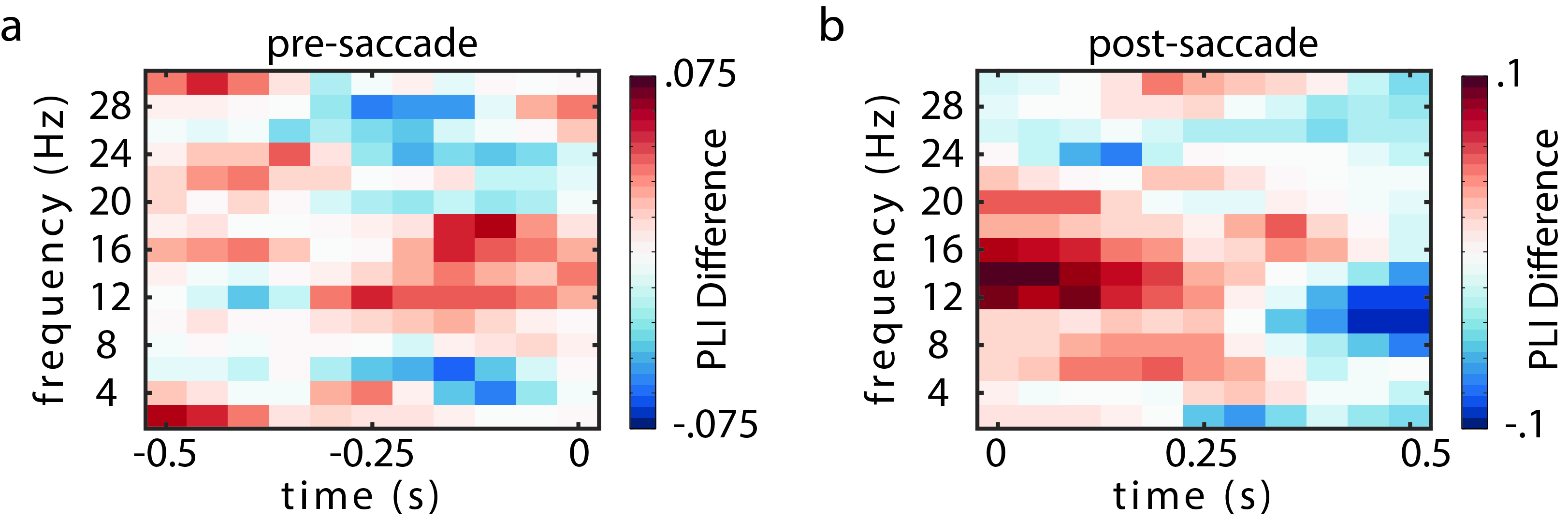

Supplement: S1 Fig — (A) Phase-locking difference (later remembered–later forgotten) on occipital depth electrodes prior to saccade offset (time = 0 s). There was no significant difference in phase locking between later-remembered and later-forgotten trials (p > 0.21; 2-sided test, fixed-effects statistics, 15 contacts in bipolar montage, time = −250 ms). (B) Phase-locking difference (later remembered–later forgotten) on occipital depth electrodes after saccade offset (time = 0 s). No significant difference in phase-locking between later-remembered and later-forgotten trials (p > 0.25; 2-sided test, fixed-effects statistics, 15 contacts in bipolar montage, time = 250 ms). Note that the statistical tests were performed on the center time bin (−250 ms and 250 ms, respectively). The data set used to generate the analyses shown in this figure can be found here: https://osf.io/tpykv. (TIF) [file pbio.2003404.s001.tif]

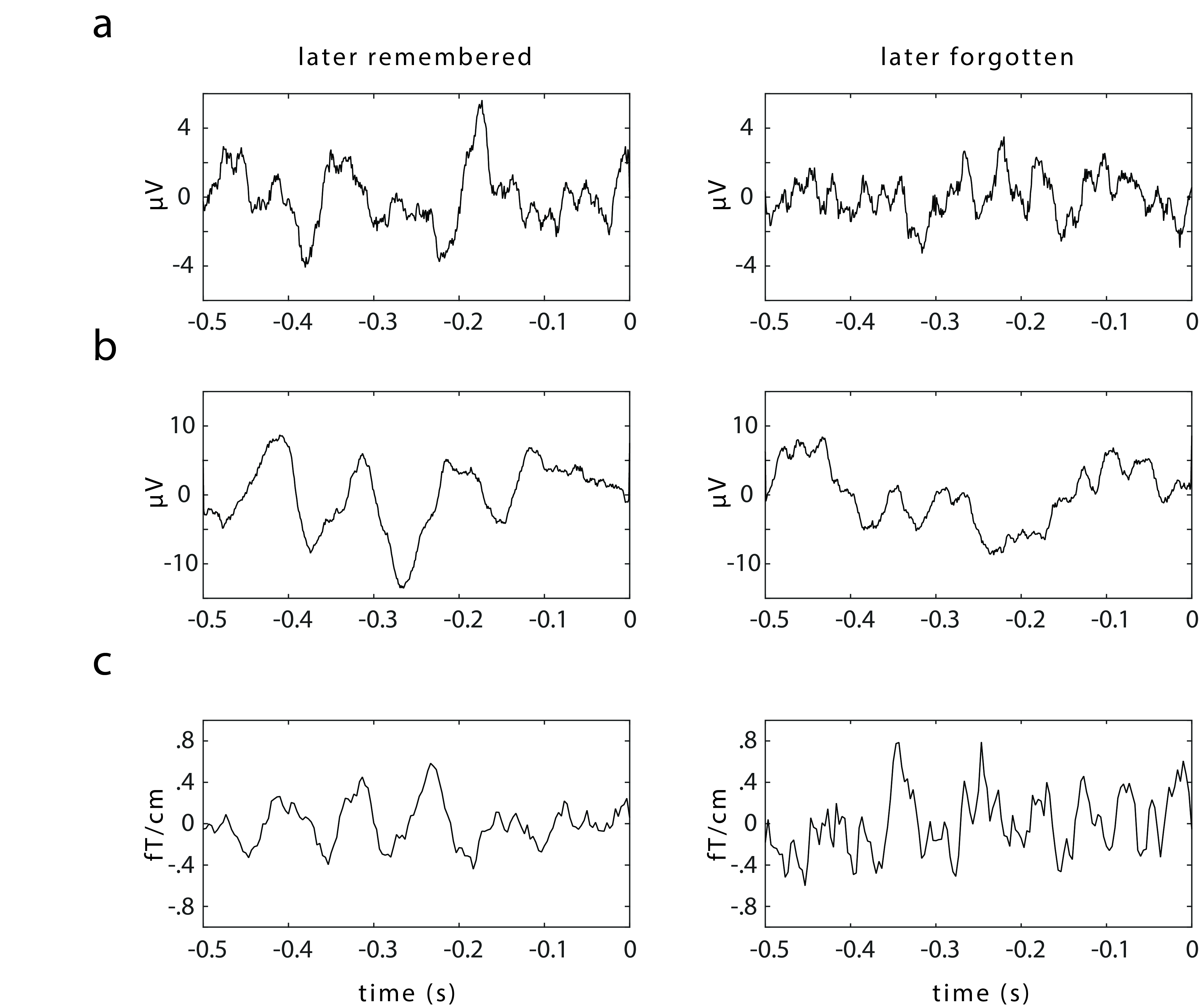

Supplement: S2 Fig — Unfiltered data, averaged across trials locked to saccade onset (time = 0 s). (A) Exemplar data from an occipital depth electrode (bipolar montage). (B) Exemplar data from a parahippocampal depth electrode (bipolar montage). (C) Exemplar data from a magnetoencephalography (MEG) sensor. Note the more regular, slow frequency fluctuations in the averaged potentials for later-remembered trials (left) as compared to later-forgotten trials (right). The data set used to generate the analyses shown in this figure can be found here: https://osf.io/tpykv. (TIF) [file pbio.2003404.s002.tif]

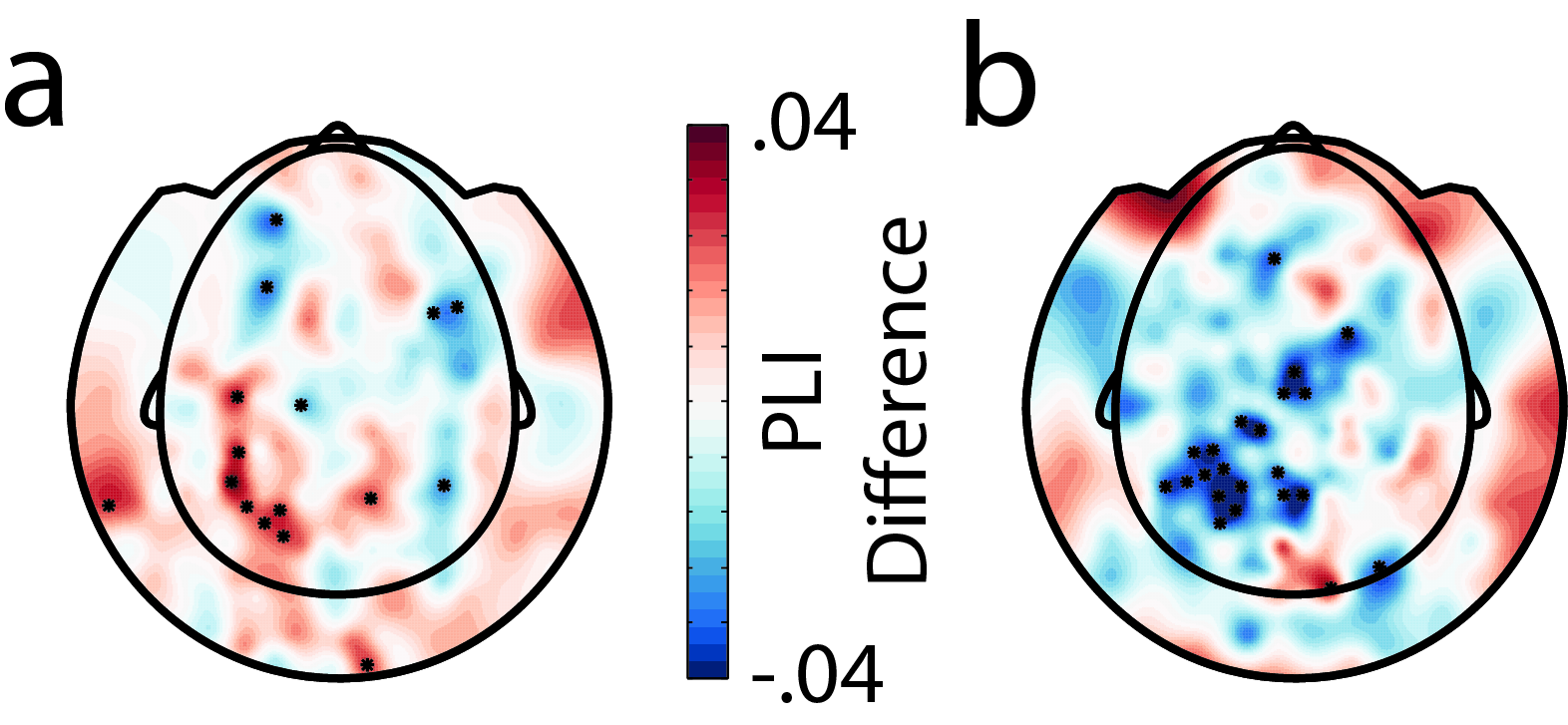

Supplement: S3 Fig — (A) Difference between later-remembered trials and surrogate data at 12–14 Hz, −250 ms. (B) Difference between later-forgotten trials and surrogate data at 12–14 Hz, −250 ms. Significant sensors are highlighted (p < 0.05, 2-sided). The data set used to generate the analyses shown in this figure can be found here: https://osf.io/tpykv. (TIF) [file pbio.2003404.s003.tif]

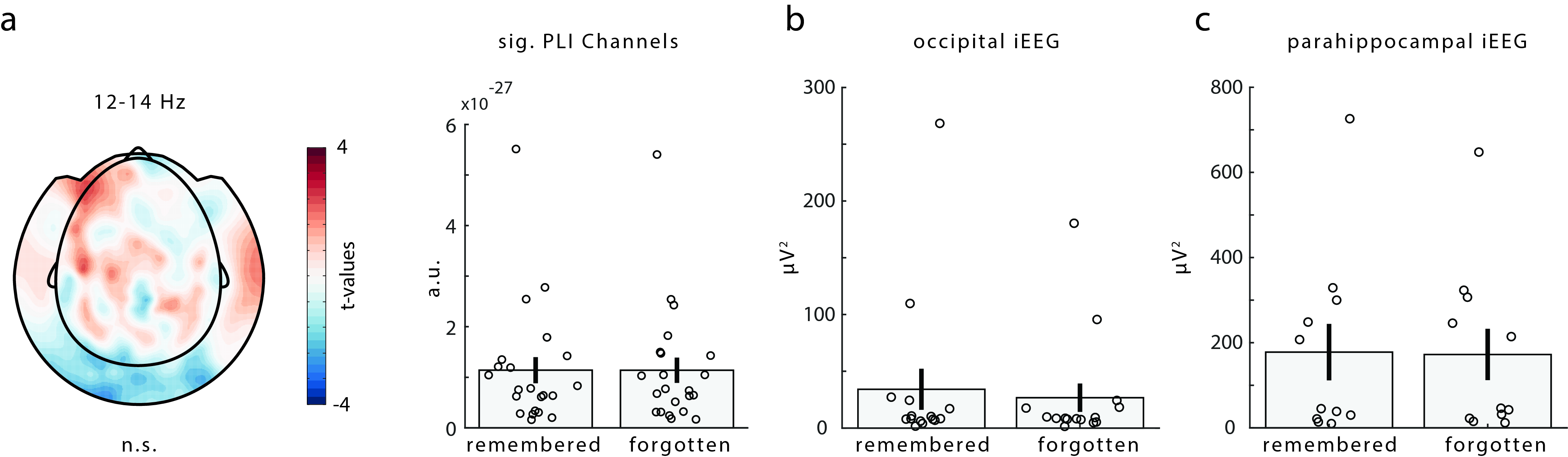

Supplement: S4 Fig — (A) Topography of magnetoencephalography (MEG) sensor-level statistics (planar gradients) for power averaged over 12–14 Hz at −0.25 s (corresponding to the phase-locking index [PLI] effect); no significant difference (cluster-based permutation statistic, no clusters found). The bar plot (error bars represent SEM; dots indicate individual participants) depicts 12–14 Hz power on sensors showing the significant phase-locking effect. No significant difference (t21 = 0.07, p > 0.9, 2-sided t test) between later-remembered and later-forgotten trials in the respective time interval (−0.25 s) was found. (B) Occipital depth electrodes showed no significant difference in power (t14 = 1.27, p > 0.2, 2-sided t test), averaged over 12–14 Hz at −0.25 s (c) Parahippocampal depth electrodes showed no significant difference in power (t10 = 0.71, p > 0.7, 2-sided t test), averaged over 8–10 Hz at −0.25 s. The data set used to generate the analyses shown in this figure can be found here: https://osf.io/tpykv. (TIF) [file pbio.2003404.s004.tif]

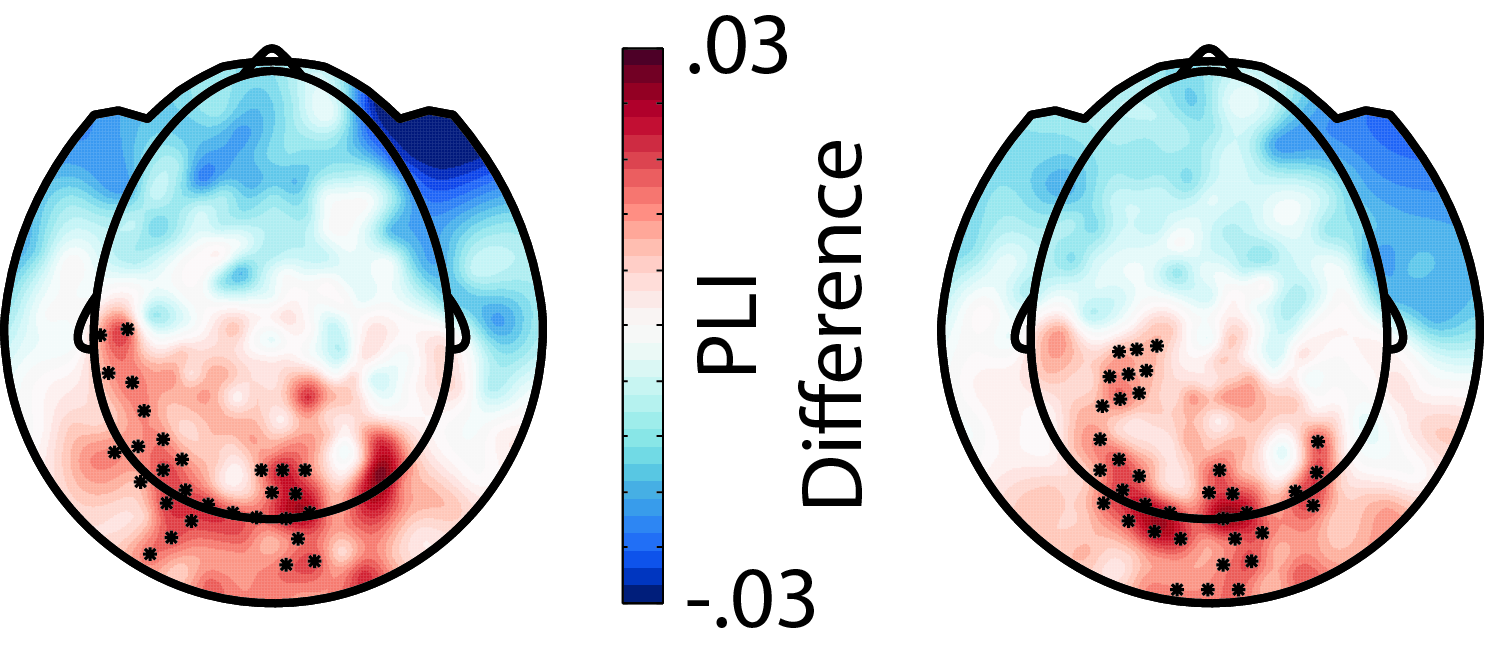

Supplement: S5 Fig — Magnetoencephalography (MEG) sensor (planar gradients) analysis shows significantly higher phase locking (phase-locking index [PLI]) for later-remembered than forgotten trials at 10 Hz (corresponding to a frequency range of 7.5 to 12.5 Hz), averaged in the −0.2- to 0-s interval prior to saccade onset (left: N = 22, p < 0.05, 2-sided test, significant sensors highlighted; right: N = 36, p < 0.005, 2-sided test, significant sensors highlighted). The data set used to generate the analyses shown in this figure can be found here: https://osf.io/tpykv. (TIF) [file pbio.2003404.s005.tif]

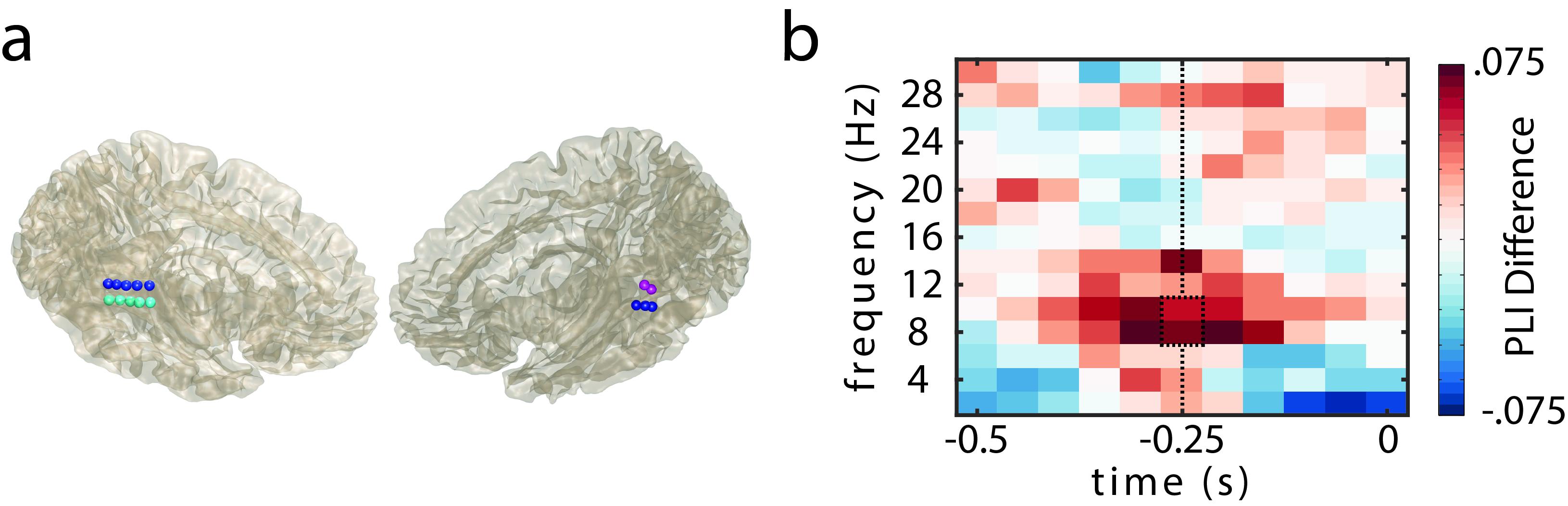

Supplement: S6 Fig — (A) Electrode locations of parahippocampal depth electrodes in 3 participants (color coded). (B) Phase-locking difference (later remembered–later forgotten) on parahippocampal depth electrodes prior to saccade onset (time = 0). Significantly higher phase locking in later-remembered versus later-forgotten trials at 8–10 Hz (p < 0.05, highlighted; 2-sided test, fixed-effects statistics, 11 contacts in bipolar montage). The data set used to generate the analyses shown in this figure can be found here: https://osf.io/tpykv. (TIF) [file pbio.2003404.s006.tif]

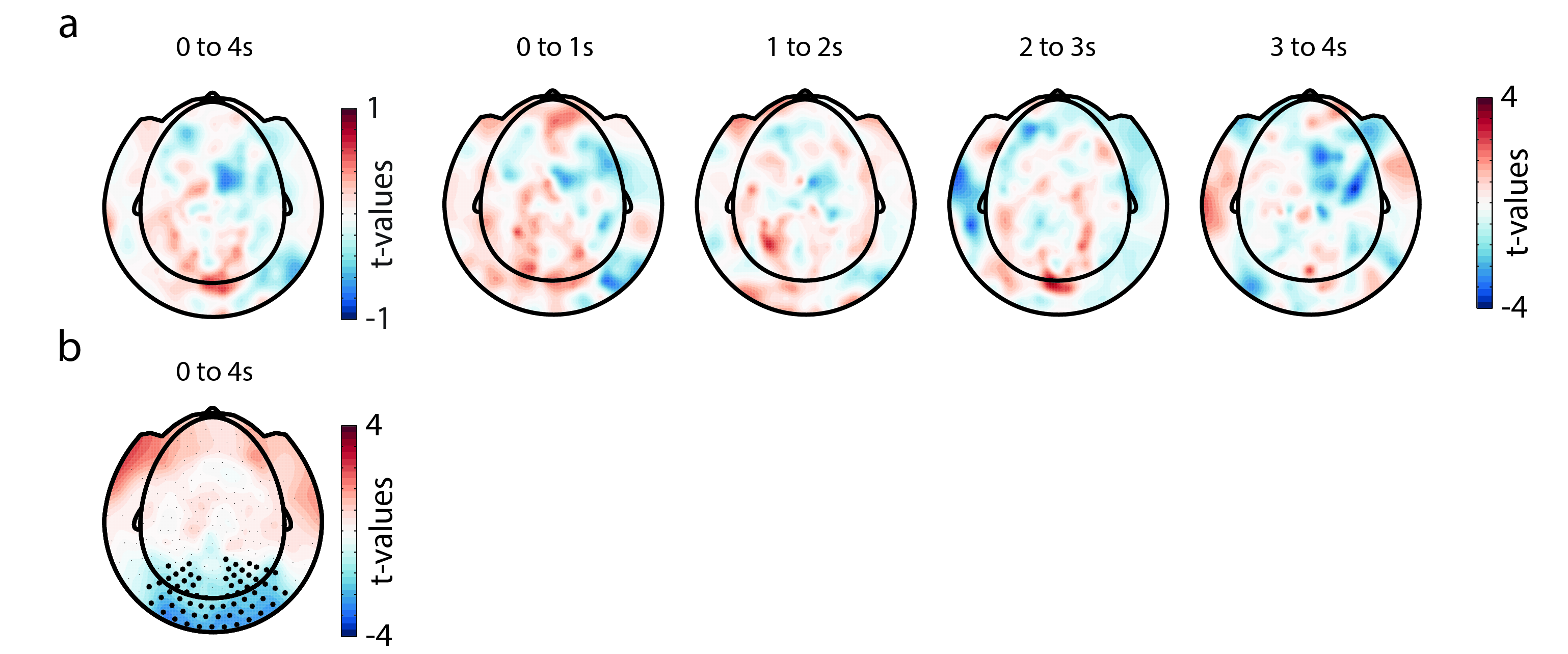

Supplement: S8 Fig — (A) Cluster-based permutation statistics showed no significant differences in phase locking between later-remembered and later-forgotten scenes at 12–14 Hz, controlling for multiple comparisons over time (0 to 4 s) and sensors (p cluster > 0.9). There were also no significant differences after averaging time over 1-s bins (all clusters p > 0.64). (B) Cluster-based permutation statistics (controlling for multiple comparisons over sensors and time: 0 to 4 s) showed significantly lower power for later-remembered scenes than for later-forgotten scenes at 12–14 Hz (p cluster < 0.018; time = 1.55–2.6 s). The data set used to generate the analyses shown in this figure can be found here: https://osf.io/tpykv. (TIF) [file pbio.2003404.s008.tif]

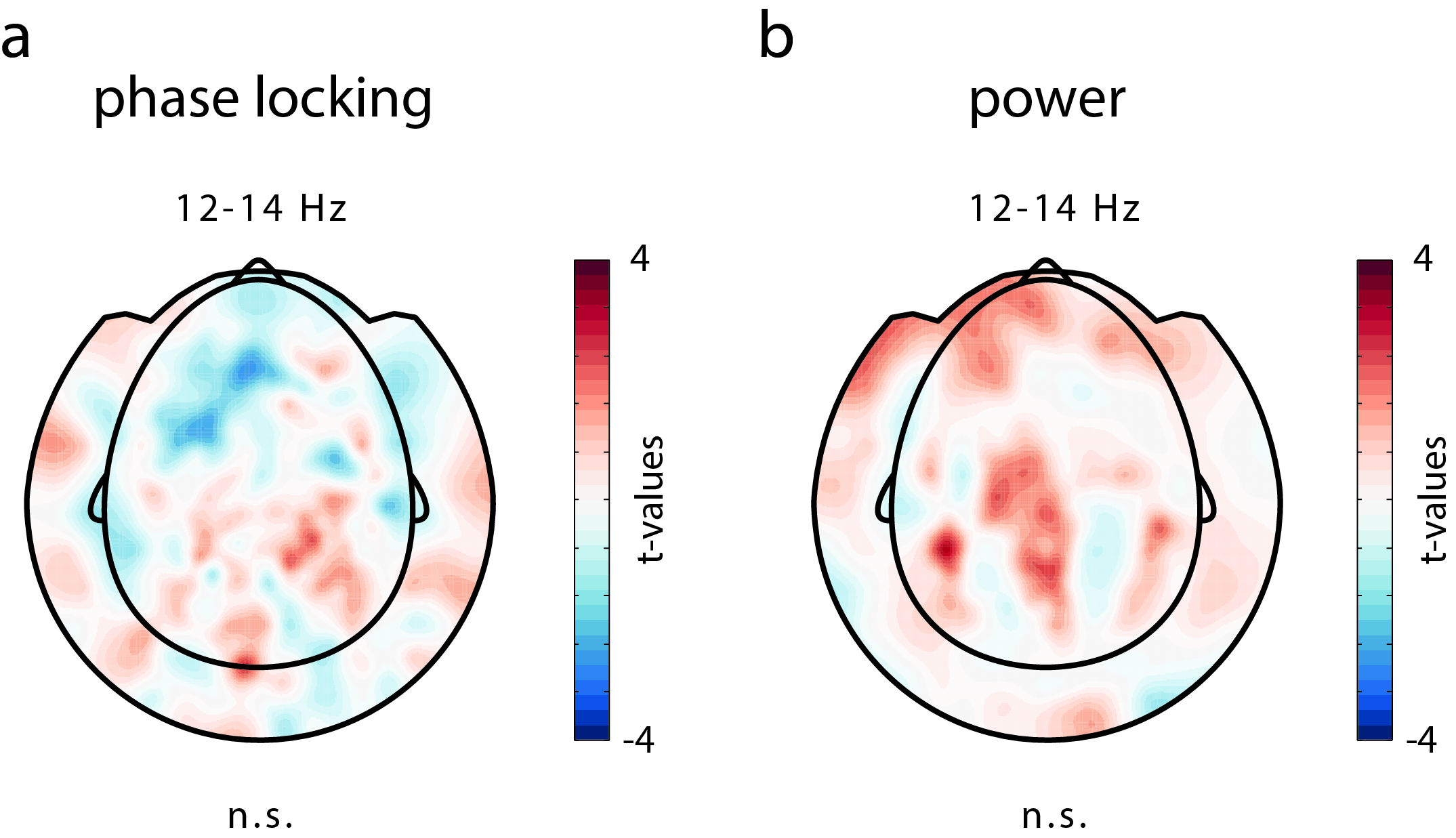

Supplement: S9 Fig — (A) Topography of magnetoencephalography (MEG) sensor-level statistics (planar gradients) for phase locking averaged over 12–14 Hz at 250 ms; no significant difference (cluster-based permutation statistic, all clusters p > 0.72). (B) Topography of MEG sensor-level statistics (planar gradients) for power averaged over 12–14 Hz at 250 ms; no significant difference (cluster-based permutation statistic, no clusters found). The data set used to generate the analyses shown in this figure can be found here: https://osf.io/tpykv. (TIF) [file pbio.2003404.s009.tif]
